# What Is Uncommon Can Be Critical: A Case of Quinolone-Induced Acute Liver Failure

**DOI:** 10.7759/cureus.14780

**Published:** 2021-04-30

**Authors:** Bibek Bakhati, Victoria M Sibi, Armugam P Mekala, Joshua A Ronen, Sai Mungara

**Affiliations:** 1 Internal Medicine, Texas Tech University Health Sciences Center School of Medicine at the Permian Basin, Odessa, USA; 2 Medicine, North-Western State Medical University, St. Petersburg, RUS

**Keywords:** acute liver failure, fulminant liver failure, quinolone toxicity, levofloxacin toxicity, n-acetylcysteine, decompensated liver failure, hepatic encephalopathy, drug-induced acute liver failure, dili, idiosyncratic drug reaction

## Abstract

Many drugs are known to potentially cause liver injury; however, only a few reports investigate the association between levofloxacin and acute liver failure (ALF).

The case describes a 65-year-old man who was admitted with primary diagnoses of cerebrovascular accident (CVA) and acute coronary syndrome (ACS) who developed an upper respiratory tract infection for which he was started on levofloxacin. Following its administration, serum aspartate aminotransferase (AST) and alanine aminotransferase (ALT) increased more than 100-fold above the upper limit of normal. Over the next 24 hours, AST peaked at 9334 U/L, ALT at 4525 U/L, prothrombin time to 24.6 seconds, international normalized ratio (INR) to 2.22, and serum ammonia to 157 µmol/L. The patient developed signs and symptoms of decompensated liver disease, namely hepatic encephalopathy (HE). Levofloxacin was discontinued immediately, and evidence-based treatment per society guidelines from The American Association for the Study of Liver Diseases consisting of IV n-acetylcysteine as well as lactulose and rifaximin was initiated. Such medical management resulted in clinical resolution of his ALF, but he had a poor overall prognosis and eventually succumbed to critical illness.

## Introduction

Acute liver failure (ALF) or fulminant hepatic failure is characterized by sudden loss of hepatic function, development of coagulopathy (international normalized ratio, INR > 1.5), and hepatic encephalopathy (HE) in a person with no known liver disease [1]. Other physical examination findings can include jaundice, vesicular skin lesions akin to that of herpes simplex virus, ascites, right upper quadrant pain, hepatomegaly, and orthostatic hypotension. The disease process is also known as acute or fulminant hepatic necrosis. In the United States, ALF is considered a rare entity with an estimate of 2000 cases each year as Lee describes [2]. A commonly used cutoff differentiating ALF from chronic liver failure is an illness duration of 26 weeks. Notably, up to 50% of cases of ALF are secondary to (dose-dependent) acetaminophen toxicity per Lee et al. [1].

Other causes include idiosyncratic (dose-independent) drug reactions (termed drug-induced liver injury or DILI), ischemia (from hypoperfusion due to cardiac insufficiency, hepatic vein thrombosis, or vasoconstricting drugs), or viral and alcoholic hepatitis; a certain number of cases still remain unspecified [1]. Aside from acetaminophen toxicity, viral hepatitis and DILI are the most common causes of ALF in adults in the United States. DILI is said to occur within six months of drug initiation (i.e. non-steroidal anti-inflammatory drugs [NSAIDs], anticonvulsants, and antibiotics).

It is worth noting that in patients known to be chronic alcoholics presenting with acute severe alcoholic hepatitis, the disease is considered acute-on-chronic liver failure even if its course has lasted less than 26 weeks. ALF is classified as hyperacute (less than seven days), acute (seven to 21 days), or subacute (three weeks to 25 weeks). Cerebral edema has been cited as a common consequence of ALF (also present in up to 75% of patients with Grade III-IV HE), while renal failure and portal hypertension have been cited as common consequences of subacute disease. Prognoses are typically better in ALF as the underlying cause is often reversible and poorer in subacute liver failure due to underlying genetic conditions (such as hereditary hemochromatosis [HHE] or Wilson's disease) or untreated viral hepatitis.

Numerous reports of ALF have been associated with fluoroquinolones as Adikwu and Deo explain [3]. However, there are only a few cases described in the literature linking ALF to levofloxacin [4-8]. The following text details a case of a patient who developed ALF following IV infusion of levofloxacin. 

## Case presentation

A 65-year-old man with a past medical history significant for hypertension, chronic obstructive pulmonary disease, and cerebrovascular accident (CVA) in 2017 with residual left-sided weakness presented to the emergency department with confusion, slurred speech, and new-onset right-sided weakness. He also had a known history of amphetamine use as well as alcohol and tobacco use. He denied any documented allergies to medications.

His vital signs on presentation were a temperature of 36.6 degrees Celsius, blood pressure of 145/105 mmHg, heart rate of 100 beats/min sinus rhythm, respiratory rate of 27 breaths/min, and an oxygen saturation of 95% on room air. On physical examination, he was alert, awake, and oriented only to person and place. Overall, he was weak and noted to be cachectic. The patient’s neurological examination was significant for aphasia, +3/5 strength in the right upper and lower extremities, +4/5 strength in the left upper and lower extremities, and a bilaterally positive Babinski sign. The laboratory results on admission showed an elevated white blood cell count (12,200/mcL), lactic acid (1.5 mmol/L), troponin (0.90 ng/mL), brain natriuretic peptide (2613.9 pg/mL), blood urea nitrogen (25 mg/dL), and creatinine (1.5 mg/dL) with normal liver function testing. An electrocardiogram showed findings suggestive of possible non ST-elevation myocardial infarction (NSTEMI). A CT scan of the head was negative for acute pathology but follow-up MRI of the brain revealed an acute medullary infarct involving the left medullary pyramid. Magnetic resonance angiogram of the brain and neck showed absence of flow within the right internal carotid artery and right middle cerebral artery. Carotid Doppler studies confirmed presence of bilateral common carotid artery stenosis. A diagnosis of CVA, NSTEMI, and acute kidney injury (AKI) was made and he was subsequently admitted to the floor for further management.

During this admission, he developed hospital-acquired right upper lobe pneumonia and was started on broad-spectrum IV antibiotics including piperacillin-tazobactam and vancomycin. Later, he was transferred to the intensive care unit as he developed acute respiratory distress syndrome and was emergently intubated as a result. Levofloxacin was also initiated for double Pseudomonal coverage.

Twenty-four hours after the initial infusion of levofloxacin he developed a predominantly hepatocellular transaminitis, evident coagulopathy, and significant hyperammonemia as detailed in the column titled "Day 4" of Table 1. Upon physical examination, no signs of jaundice or hepatomegaly were present. On neurological evaluation, he was responsive only to noxious stimuli. The patient remained critically ill and started to deteriorate further. Therapy consisting of IV n-acetylcysteine infusion protocol, lactulose, and rifaximin was initiated. It was suspected that his symptoms and laboratory findings represented fluoroquinolone-induced ALF; therefore, levofloxacin was stopped immediately. Over the following days, signs and symptoms of his decompensated liver disease in the setting of ALF improved including some improvement in mentation. However, his prognosis was poor. He developed multi-system organ failure and unfortunately expired shortly after. 

**Table 1:** Trajectory of liver function test before and after administration as well as discontinuation of levofloxacin AST, aspartate aminotransferase; ALT, alanine aminotransferase; ALP, alkaline phosphatase; PT, prothrombin time; INR, international normalized ratio. IV levofloxacin was started on Day 3 and discontinued immediately upon recognition of acute liver failure on Day 4.

	Reference values	Hospitalization							
		Admission day	Day 2	Day 3	Day 4	Day 5	Day 6	Day 7	Day 8
AST	3-38 U/L	28	51	93	9334	3684	2671	835	271
ALT	≤49 U/L	14	13	58	4525	3294	2445	1680	1127
ALP	41-107 U/L	59	41	44	52	69	71	80	85
Total bilirubin	0.1-1.2 mg/dL	0.7	0.7	0.9	1.5	0.9	0.9	0.6	0.6
PT	12.2-14.9 seconds	14.6	-	-	24.6	24.3	21.1	21.0	21.2
INR		1.14	-	-	2.22	2.18	1.82	1.82	1.83
Ammonia	16-52 umol/L	-	-	-	157	110	76	54	54

## Discussion

The presentation of markedly elevated serum transaminases, coagulopathy, and HE in a patient without any prior history of liver disease is necessary to make the diagnosis of ALF [1]. The most common drug-induced cause of ALF is acetaminophen, followed by antibiotics as Chang and Schiano describe [9].

Levofloxacin, a third-generation fluoroquinolone antibiotic, is used in the treatment of a variety of bacterial infections. It is a generally a well-tolerated drug with a good safety profile [3,10]. Although infrequent, it has been linked to incidences of ALF as Hoofnagle et al. point out [3,10]. The mechanism of levofloxacin-induced liver injury is still not clear, and the severity ranges from transient to mild elevation of liver function tests to ALF [10].

Based on the pattern of serum liver enzyme elevations, liver injury itself can be categorized into hepatocellular, cholestatic, or mixed hepatocellular-cholestatic [10]. To indicate whether the type of injury is hepatocellular, cholestatic, or mixed, the R-factor is applied. R is equal to alanine aminotransferase (ALT)/upper limit of normal (ULN) divided by ALP/ULN. When the R-factor is greater than or equal to 5, it is suggestive of hepatocellular liver injury. A value of less than 2 demonstrates a cholestatic type of injury, while a mixed hepatocellular-cholestatic injury is indicated when R-factor is greater than 2 but less than 5 as Chalasani et al. elaborate [11]. As displayed in Table 1, the patient’s laboratory values included an ALT of 4525 (ULN = 49) and an ALP of 52 (ULN = 107), yielding an R-factor of 190 suggestive of a hepatocellular pattern of liver injury. For reference, patterns of liver injury in acetaminophen toxicity, ischemic hepatitis, alcoholic hepatitis, and hepatitis B virus can yield the following (respectively): aminotransferases >3500 IU/L with low bilirubin and high INR, aminotransferases 25-250 times the ULN and elevated serum lactate dehydrogenase, aspartate aminotransferase (AST) typically two to three times greater than ALT, and aminotransferases between 1000 and 2000 IU/L with ALT being greater than AST. In this case, the patient developed acute liver injury a day following initiation of levofloxacin therapy. This is consistent, per Orman et al., with the short latency period (time to onset) of hepatic injury that is seen with fluoroquinolones [10-12].

There are two mechanisms of liver injury associated with drugs: predictable or unpredictable (idiosyncratic) [10]. The first mechanism is dose-dependent (i.e. acetaminophen) and is due to direct toxicity of the drug or its metabolite. The latter, however, has no relation to the dose of the drug as Roth and Ganey specify (dose-independent, i.e. NSAIDs, antibiotics, and anticonvulsants) [13]. The drug reaction in the case described is idiosyncratic, as are the majority cases of DILI per Hussaini and Farrington [14].

Other parameters to diagnose ALF include evidence of decompensated liver disease, such as HE and coagulopathy, as well as additional physical examination findings including but not limited to jaundice, vesicular lesions, asterixis, right upper quadrant pain, abdominal distension from ascites, and hepatomegaly. The patient’s clinical presentation was indicative of Grade III HE. There was notable improvement in liver function testing in the days following discontinuation of levofloxacin with ongoing supportive care, further supporting the diagnosis of quinolone-induced ALF.

## Conclusions

The diagnosis of ALF is confirmed with the presence of liver injury, coagulopathy, and HE in addition to other physical examination findings. Illness duration plays a factor in determining the cause, whether hyperacute, acute, or subacute. The delineation between acute and chronic disease occurs at 26 weeks. Notably, severe acute alcoholic hepatitis is often considered to represent acute-on-chronic liver failure due to the likely prolonged history of alcohol abuse in affected patients. Reversible causes of acute liver injury, such as ischemia (due to cardiac insufficiency, veno-occlusive disease, or illicit vasoconstricting substances), viral hepatitis, and alcohol hepatitis, tend to have a better prognosis and be hyperacute or acute as it pertains to their illness duration. Irreversible causes in patients with genetic predisposition to diseases like HHE or Wilson's disease tend to have a poorer prognosis and be subacute or even chronic illness duration-wise with risks of liver cirrhosis looming in the background. This group could be living with undetected disease for much of their lifetime. Another important factor to also clarify is whether the mechanism of liver injury is predictable or not (i.e. idiosyncratic or DILI), as the question of dose dependency helps narrow down possible differential diagnoses. Although the incidence of levofloxacin-induced ALF is exceedingly rare, a high index of suspicion is necessary to make the diagnosis, discontinue the offending agent, and initiate clinically appropriate evidence-based medical therapy to preserve morbidity and mortality.
